# Clinical application of sodium-glucose cotransporter 2 inhibitor into a real-world setting of heart failure care

**DOI:** 10.1186/s12933-020-01113-5

**Published:** 2020-09-02

**Authors:** Atsushi Tanaka, Koichi Node

**Affiliations:** Department of Cardiovascular Medicine, Saga University, 5-1-1 Nabeshima, Saga, 849-8501 Japan

**Keywords:** Type 2 diabetes, Sodium glucose co-transporter 2 inhibitor, Heart failure, Clinical outcome trial, Real-world

## Abstract

For years there have been concerns whether the results of large-scale clinical trials that include limited specific patient populations can be applied to patients in real-world clinical practice. Therefore, it is crucially important to verify whether emerging evidences obtained from large-scale clinical trials on limited specific patient populations can be applied to patients at real-world clinical settings. Recent cardiovascular outcome trials with sodium-glucose cotransporter 2 (SGLT2) inhibitors showed a consistent risk reduction of approximately 30% for hospitalization for heart failure (HF), and the SGLT2 inhibitors had a great potential to be effective for prevention of HF in a wide variety of type 2 diabetes (T2D) patients independent of their history of HF or cardiovascular disease (CVD). Furthermore, the DAPA-HF trial also demonstrated that dapagliflozin proved clinically effective in patients with HF with reduced ejection fraction regardless of diabetes, suggesting its robust benefits in some specific patients with HF. According to these evidences, SGLT2 inhibitor is increasingly recognized as an emerging and promising option to reduce the risk of HF in patient with T2D. To use appropriately SGLT2 inhibitors for HF prevention in the real-world setting, it would be required to determine the optimal patient population who can receive better clinical benefits from SGLT2 inhibitors. In this commentary, based on the current understandings and lessons learned from the most recent studies, we discussed the importance of future research on the safety and efficacy of SGLT2 inhibitor in clinical situations of HF other than those examined in previous cardiovascular outcome trials.

For years there have been concerns whether the results of large-scale clinical trials that include limited specific patient populations can be applied to patients in real-world clinical practice. When the clinical background and characteristics of a patient match those of a ‘specific’ patient population in a clinical trial it is essential to know whether the patient can achieve similar benefits to those observed in the trial, beyond consideration of the estimated number needed to treat. It is therefore important to verify whether clinical trial results can be applied to patients at a general population level by evaluating real-world data and using an appropriate estimation model. This is an important step when aligning daily medical care with guidelines that are formulated based mainly on the results of large-scale clinical trials.

Recent cardiovascular outcome trials (CVOTs) on newer antidiabetic agents have increasingly resulted in major paradigm shifts in care aimed at preventing cardiovascular and renal complications in patients with type 2 diabetes (T2D). Among those agents, a sodium-glucose cotransporter 2 (SGLT2) inhibitor is one class of glucose-lowering agents, which acts uniquely via inhibition of glucose reabsorption in the renal proximal tubule. Yet, a meta-analysis of previous CVOTs for SGLT2 inhibitor highlighted the utility of SGLT2 inhibitor in preventing and/or delaying the development of heart failure (HF) in patients with type 2 diabetes (T2D) [1]. More recently, the eValuation of ERTugliflozin EffIcacy and Safety CardioVascular Outcomes Trial (VERTIS-CV) also demonstrated that ertugliflozin reduced the risk of hospitalization for HF in patients with T2D and atherosclerotic CVDs [2], suggesting that beneficial effect of SGLT2 inhibitors on HF outcome was consistent across the CVOTs. In a treatment algorithm in patients with T2D and high cardiovascular risk, SGLT2 inhibitor was recommended to reduce the risks of HF, cardiovascular events, and death [3]. However, the proportion of individuals with a history of HF was substantially limited in previous CVOTs [4]. Additionally, the subjects’ cardiac phenotypes of HF were not fully identified. Therefore, there has been an urgent need to assess whether SGLT2 inhibitor can be beneficial in patients with overt HF and for which phenotype of HF the agent can be clinically useful.

A previous large observational study suggested that compared to other glucose-lowering drugs initiation of SGLT2 inhibitor was associated with a lower risk of death and HF in real-world patients with T2D regardless of pre-existing CVDs [5]. Recently, Bassi et al. [6] reported intriguing results obtained from a population level study in the USA that used a decision-analytic model to quantify the extrapolated burden of deaths prevented or postponed by optimal implementation of SGLT2 inhibitor therapy in HFrEF patients. Despite several methodological limitations this study highlights the incremental benefits of SGLT2 inhibitor added to guideline-directed HF-therapy in that patient population. Because of frequent incompleteness of guideline-directed HF-therapies in real-world clinical setting [7], however, it is necessary to determine whether the use of SGLT2 inhibitor before completion of guideline-directed HF-therapies is also beneficial. Furthermore, it is necessary to clarify the detailed patient population that can optimally gain clinical benefits from SGLT2 inhibitor therapy in real-world settings.

In 2019, the Dapagliflozin and Prevention of Adverse outcomes in Heart Failure (DAPA-HF) trial was the first to show that dapagliflozin treatment reduced the risk of hospitalization for HF and mortality specifically in patients with HF with reduced ejection fraction (HFrEF) regardless of T2D [8]. In a recent post hoc analysis using data obtained from that trial, a consistent clinical benefit of dapagliflozin was observed irrespective of established HF therapies and drug treatments [9]. This suggests that dapagliflozin, in addition to various types of guideline-based treatments for HF, had incremental and complementary therapeutic effects in patients with HFrEF. These findings could expand the clinical versatility of dapagliflozin in HFrEF care and will greatly affect the next revision of guidelines for the treatment of HFrEF.

Given these findings and the current therapeutic algorithm for symptomatic HFrEF patients in guidelines, the use of dapagliflozin may be useful in preventing and/or delaying the need for downstream therapies. Earlier treatment with dapagliflozin in patients with symptomatic HFrEF may have a positive impact on outcomes. Nonetheless, whether dapagliflozin would be an effective replacement in some clinical settings of up-titrated standard drugs (incl. angiotensin-converting enzyme inhibitor/β-blocker/mineralocorticoid receptor antagonist) and considering additional therapies, such as cardiac device, is still uncertain. Additionally, clinical efficacy of SGLT2 inhibitor is unknown in several specific HF conditions, such as drug-naïve patients, electrical disorders-derived, severely reduced ejection fraction, New York Heart Association class IV, and acute decompensated situation, since those patients were generally excluded or were a minor population in CVOTs. Additionally, cardiologists should even expect the possible application of SGLT2 inhibitor in patients with HF with preserved ejection fraction.

Taken together, SGLT2 inhibitor is increasingly recognized as an emerging and promising treatment option to reduce the risk of cardiovascular events, including HF, in patient with T2D [10]. Due to its efficacy and beneficial impact on HF-related outcomes, drug-repositioning of SGLT2 inhibitor could be critical in resolving the unmet needs of HF care. Further research is therefore warranted to strengthen the clinical utility of SGLT2 inhibitor in a broad range of real-world clinical settings in patients with HF. At the same time, explorations for its mode of action against HF beyond glucose-lowering should become another research topic of great interest [11].

## Data Availability

Not applicable.
